# Cellular localization of NRF2 determines the self-renewal and osteogenic differentiation potential of human MSCs via the P53–SIRT1 axis

**DOI:** 10.1038/cddis.2016.3

**Published:** 2016-02-11

**Authors:** D S Yoon, Y Choi, J W Lee

**Affiliations:** 1 Department of Orthopaedic Surgery, Yonsei University College of Medicine, Seoul, South Korea; 2 Brain Korea 21 PLUS Project for Medical Science, Yonsei University, Seoul, South Korea

## Abstract

NRF2 (nuclear factor erythroid-derived 2-like 2) plays an important role in defense against oxidative stress at the cellular level. Recently, the roles of NRF2 in embryonic and adult stem cells have been reported, but its role in maintaining self-renewal and differentiation potential remains unknown. We studied the mechanisms of NRF2 action in mesenchymal stem cells (MSCs) derived from human bone marrow. We found that the cellular localization of NRF2 changed during prolonged cell passage and osteogenic differentiation. Blocking the nuclear import of NRF2 using ochratoxin A (OTA) induced the loss of the self-renewal and osteogenic potential of early-passage (EP) MSCs. Conversely, reinforcing the nuclear import of NRF2 using *tert*-butylhydroquinone (t-BHQ) improved the self-renewal capacity and maintained the differentiation potential in the osteogenic lineage of EP MSCs. Real-time quantitative PCR and western blot analysis showed that NRF2 positively regulates sirtuin 1 (SIRT1) at the mRNA and protein levels via the negative regulation of p53. The self-renewal and osteogenic potential suppressed in OTA-treated or NRF2-targeting small hairpin RNA (shRNA)-infected EP MSCs were rescued by introducing small interfering RNA (siRNA) targeting p53. t-BHQ treatment in late-passage (LP) MSCs, which lost their self-renewal and osteogenic potential, reversed these effects. In LP MSCs treated with t-BHQ for ∼7 days, the phosphorylation and nuclear localization of NRF2 improved and SIRT1 protein level increased, whereas p53 protein levels decreased. Therefore, our results suggest that NRF2 plays an important role in regulating p53 and SIRT1 to maintain MSC stemness. This study is the first to establish a functional link between NRF2 and SIRT1 expression in the maintenance of MSC self-renewal and differentiation potential.

Mesenchymal stem cells (MSCs) grown in *ex vivo* conditions become senescent during prolonged cell passage. Consequently, long-term cultured stem cells are not used for tissue regeneration because they do not function as stem cells after reaching therapeutically effective amounts during *in vitro* cultivation. Appropriate culture methods are very important to maintain MSC stem cell potential because their self-renewal and multipotency decrease during *ex vivo* cultivation.^1, 2, 3, 4, 5^ The decrease in the self-renewal and differentiation potentials during *ex vivo* conditions may be related to exposure to exogenous oxidative stress or to reactive oxygen species (ROS) that are endogenously generated in *in vitro* cultured cells.^6, 7^ Hypoxic conditions have been extensively examined with respect to the maintenance of the self-renewal and multipotency of human MSCs during long-term cultivation.^8, 9, 10, 11^ Indeed, *in vivo* MSC niches where the cells reside exhibit low-oxygen tension (2–8%).^12^ A recent study has also reinforced the importance of hypoxic conditions for the maintenance of MSC stemness.^13^ Therefore, hypoxic conditions may be essential for the maintenance of the early characteristics of *ex vivo* cultured MSCs. In hypoxic environments, the osteogenic and adipogenic potentials of human bone marrow-derived MSCs (BM-MSCs) decrease.^13^ However, their self-renewal capacity is maintained.^14, 15^ These results indicate that hypoxic environments in MSC cultivation maintain the undifferentiated state of MSCs without affecting their differentiation potential, promoting stemness over differentiation. However, no studies have examined the master regulators related to the maintenance of stemness in MSC cultivation in hypoxic environments *in vitro*.

Nuclear factor erythroid-derived 2-like 2 (NRF2) plays an important role in defense against oxidative stresses.^16, 17^ It induces the expression of antioxidant genes that protect many cell types from oxidative stress caused by tissue damage, such as inflammation and injury.^18^ Under normal condition, NRF2 remains in the cytoplasm, and is degraded via ubiquitination triggered by binding with kelch like-ECH-associated protein 1 (KEAP1).^19^ Under oxidative stress, NRF2 is phosphorylated and moves from the cytoplasm to the nucleus by disrupting the complex with KEAP1.^20, 21^ In this regard, the regulation of NRF2 cellular localization may be important to overcome MSC aging during prolonged cell passages *in vitro* because oxidative stress induces premature senescence of MSCs.^22^ NRF2 overexpression protects MSCs against cell death and apoptosis caused by oxidative stress and retains multi-differentiation potential.^23^ A recent study has shown that NRF2 controls the self-renewal and pluripotency of human embryonic stem cells.^24^ These results imply that NRF2 may regulate MSC stemness. However, no studies to date have clarified the relationship between NRF2 and MSC stemness or interactions between NRF2 and stemness genes. Enhancing NRF2 activity may mimic the effects of hypoxic environments, similar to an *in vivo* MSC niche in *ex vivo* cultured MSCs.

Here, we identify NRF2 as a regulator of sirtuin 1 (SIRT1). NRF2 phosphorylation was decreased and NRF2 was exported from the nucleus in late-passage (LP) and differentiated MSCs. The inhibition of NRF2 activity in early-passage (EP) MSCs suppressed the self-renewal and differentiation capacities. NRF2 activation in EP or LP MSCs enhanced the self-renewal capacity, but suppressed the differentiation potential. NRF2 increased SIRT1 protein expression by decreasing p53 protein levels. RNA interference targeting p53 in NRF2-knockdown MSCs rescued SIRT1 protein levels as well as the self-renewal and differentiation potentials. Our data indicate that NRF2 plays an important role in the maintenance of MSC stemness via p53–SIRT1 regulation.

## Results

### NRF2 phosphorylation and activity decreases during long-term cultivation and differentiation in human BM-MSCs

**Figure 1:**
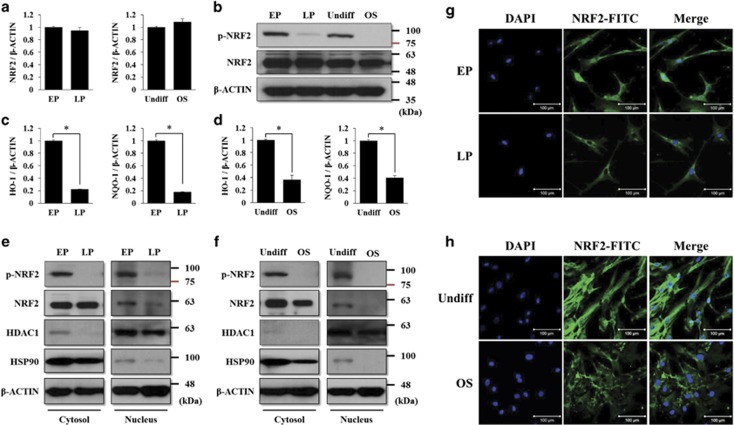
The phosphorylated level and nuclear localization of NRF2 decrease during prolonged cell passages and osteogenic differentiation. (**a**) EP, LP, undifferentiated, or differentiated MSCs to the osteogenic lineage were harvested at each stage to prepare cell lysates. The mRNA expression of *NRF2* was analyzed by qRT-PCR, and (**b**) the protein levels of NRF2 and phosphorylated NRF2 were analyzed by western blot analysis. The expression level of *β*-CATENIN was used as a loading control. For the same cDNA, the mRNA expression levels of *HO-1* (**c**) and *NQO-1* (**d**), which are downstream targets of NRF2, were also analyzed by qRT-PCR. *P<0.05 compared with control EP or undifferentiated MSCs. (**e**) In the same conditions, the cell lysates were prepared and fractionated into nuclear and cytosolic extracts according to the manufacturer's instructions. The protein levels of NRF2 and phosphorylated NRF2 were analyzed by western blot analysis in the EP and LP MSCs (**e**) or undifferentiated or osteogenic (OS)-differentiated MSCs (**f**). The protein level of HSP90 was used as a loading control for cytosolic extracts, and the protein level of HDAC1 was used as a loading control for nuclear extracts. Immunofluorescence was performed to observe the nuclear and cytosolic localization of NRF2 in the EP and LP MSCs (**g**) or undifferentiated or OS-differentiated MSCs (**h**). The nucleus was stained with DAPI, and NRF2 was stained with FITC-conjugated secondary antibody. The images were obtained using confocal microscopy. Scale bar=100 *μ*m. EP, early-passage (passages 1–3) MSCs; LP, late-passage (passages 7–10) MSCs; OS, MSCs differentiated to the osteogenic lineage for 10 days; Undiff, undifferentiated MSCs

We hypothesized that NRF2 expression or activity decrease during the long-term cultivation or osteogenic differentiation of MSCs. Accordingly, we observed the mRNA and protein levels of NRF2 in EP or LP MSCs as well as in those that were undifferentiated or differentiated to the osteogenic lineage. As shown in Figures 1a and b, the mRNA and protein levels of NRF2 did not change during prolonged cell passage or osteogenic differentiation. The nuclear import of NRF2 is blocked by its dephosphorylation, resulting in a decrease in NRF2 activity.^25^ Consistent with this, we found that the levels of NRF2 phosphorylation decreased during these processes (Figure 1b, upper panel). We also confirmed that the mRNA levels of *HO-1* (*heme oxygenase-1*) and *NQO-1* (*NAD(P)H dehydrogenase (quinone 1)*), which are downstream target genes of NRF2, decreased during these processes (Figures 1c and d). Next, the cellular localization of NRF2 and its phosphorylated form were analyzed by cytosolic and nuclear fractionated western blots and immunocytochemistry. The protein levels of nuclear and phosphorylated NRF2 were nearly detected in LP or differentiated MSCs compared with EP or undifferentiated MSCs (Figures 1e and f). Immunocytochemistry verified the absence of nuclear NRF2 in the LP and differentiated MSCs (Figures 1g and h). These results indicate that NRF2 phosphorylation and activity may be important factors in the maintenance of the early characteristics of MSCs, whereas the mRNA and protein levels did not change during prolonged cell passages and the osteogenic differentiation process. It is also thought that a decrease in NRF2 activity may be related to loss of EP MSC stemness during these processes. LP MSCs lose their antioxidant ability, which is accompanied by NRF2 dephosphorylation, resulting in higher susceptibility to oxidative stresses than that of EP MSCs.

### Regulation of NRF2 activity affects the self-renewal and osteogenic differentiation potentials of EP MSCs

**Figure 2:**
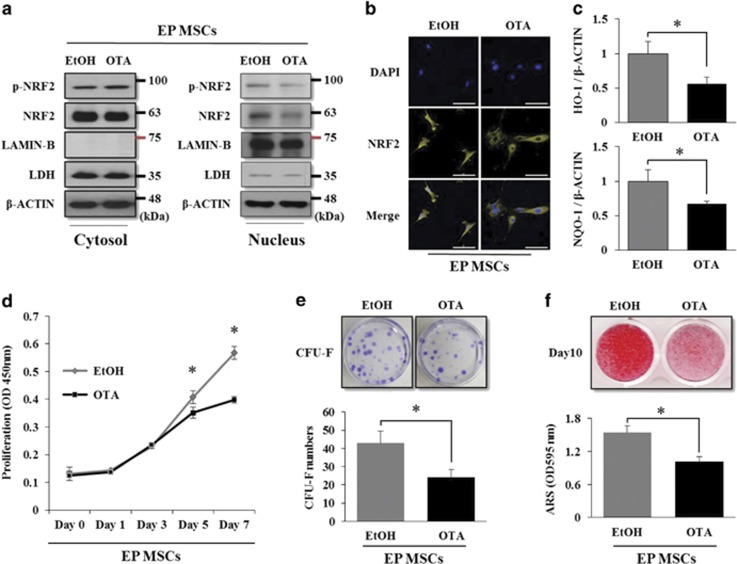
OTA induces nuclear export of NRF2 and decreases the self-renewal capacity and osteogenic differentiation in EP MSCs. (**a**) EP-MSCs were incubated in basal growth medium (DMEM-LG containing 10% FBS) in the presence of EtOH or OTA (10 *μ*M) for 16 h. The cell lysates were prepared for EtOH-treated or OTA-treated EP MSCs, and then fractionated into nuclear and cytosolic extracts. The protein levels of NRF2 and phosphorylated NRF2 were analyzed by western blot analysis for EtOH-treated or OTA-treated EP MSCs. The protein level of LDH was used as a loading control for cytosolic extracts, and the protein level of LAMIN-B was used as a loading control for nuclear extracts. The expression level of *β*-CATENIN was also used as a loading control for both cytosolic and nuclear extracts. EtOH was used as a vehicle for OTA. (**b**) Immunofluorescence was performed to observe the nuclear and cytosolic localization of NRF2 in the EtOH-treated or OTA (10 *μ*M)-treated EP MSCs. The nuclei were stained with DAPI, and NRF2 was stained with Alexa Fluor 568 (Yellow)-conjugated secondary antibody. The images were obtained using confocal microscopy. Scale bar=100 *μ*m. (**c**) The mRNA expression levels of HO-1 and NQO-1 were also analyzed by qRT-PCR. *P<0.05 compared with control EtOH-treated MSCs. (**d**) A cell proliferation assay was performed to determine the proliferative capacities of EtOH- or OTA (10 *μ*M)-treated EP MSCs using an EZ-Cytox Kit. Each experiment was performed in triplicate (*n*=3). (**e**) EP-MSCs (1 × 10^3^ cells per well in 100-mm dishes) treated with EtOH or OTA (10 *μ*M) were incubated in basal growth medium for 12 days. The colony-forming abilities were compared for EP MSCs treated with EtOH or OTA using crystal violet (CV) staining, and the numbers of colony-forming cells were counted in triplicate by three observers (*n*=3). *P<0.05 compared with EtOH-treated EP MSCs. (**f**) EP MSCs (8 × 10^4^ cells per well in 12-well plates) treated with EtOH or OTA (10 *μ*M) were incubated in osteogenic medium for 10 days. Alizarin red S staining was performed to detect mineral deposition at day 10. For quantitative analysis, absorbance was measured at 595 nm following destaining with 10% cetylpyridinium for 30 min. *P<0.05 compared with EtOH-treated EP MSCs

To confirm whether NRF2 activity affects the self-renewal and differentiation potentials of MSCs, ochratoxin A (OTA), which is a NRF2 inhibitor,^26^ was used to suppress NRF2 activity in EP MSCs. Treatment with 10 *μ*M OTA successfully suppressed the nuclear import of NRF2 and its phosphorylated form (Figure 2a). Immunofluorescence also showed the suppression of nuclear import (Figure 2b), and the mRNA levels of *HO-1* and *NQO-1* decreased in response to OTA treatment (Figure 2c). To assess the effects of OTA on the self-renewal capacity of EP MSCs, proliferation and colony-forming unit fibroblast (CFU-F) assays were performed. There was no difference in the proliferation rates between control EP MSCs and OTA-treated EP MSCs at day 3, but the gap between the two groups gradually widened up to day 7 (Figure 2d). OTA significantly reduced the colony-forming ability of EP MSCs cultured for 12 days (Figure 2e) and blocked the ability to differentiate into the osteogenic lineage (Figure 2f). To exclude the nonspecific effects of the compounds, small interfering RNA (siRNA) knockdown of NRF2 and its effects on self-renewal and differentiation of EP MSCs were evaluated, thus resulting in the same effects as OTA treatment (Supplementary Figure S1). These results showed that the self-renewal and differentiation ability of EP MSCs are inhibited by blocking the nuclear import and phosphorylation of NRF2.

**Figure 3:**
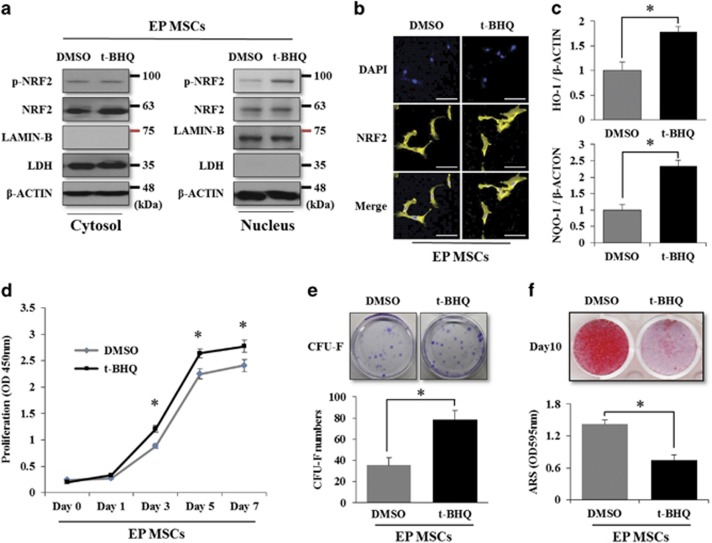
t-BHQ induces nuclear import of NRF2 and enhances the self-renewal capacity, but does not affect the osteogenic differentiation of EP MSCs. (**a**) EP MSCs were incubated in basal growth medium (DMEM-LG containing 10% FBS) in the presence of DMSO or t-BHQ (10 *μ*M) for 16 h. Cell lysates were prepared for the DMSO-treated or t-BHQ-treated EP MSCs, and then fractionated into nuclear and cytosolic extracts. The protein levels of NRF2 and phosphorylated NRF2 were analyzed by western blot analysis for the DMSO- or t-BHQ-treated EP MSCs. The LDH protein level was used as a loading control for cytosolic extracts, and the protein level of LAMIN-B was used as a loading control for nuclear extracts. The expression level of *β*-CATENIN was also used as a loading control for both cytosolic and nuclear extracts. DMSO was used as a vehicle of t-BHQ. (**b**) Immunofluorescence was performed to observe the nuclear and cytosolic localization of the NRF2 protein in the DMSO- or t-BHQ (10 *μ*M)-treated EP MSCs. The nuclei were stained with DAPI, and NRF2 was stained with Alexa Fluor 568 (Yellow)-conjugated secondary antibody. The images were obtained using confocal microscopy. Scale bar=100 *μ*m. (**c**) The mRNA expression levels of *HO-1* and *NQO-1* were also analyzed with qRT-PCR. *P<0.05 compared with control DMSO-treated MSCs. (**d**) A cell proliferation assay was performed to determine the proliferative capacities of DMSO- or t-BHQ (10 *μ*M)-treated EP MSCs using an EZ-Cytox Kit. Each experiment was performed in triplicate (*n*=3). (**e**) EP-MSCs (1 × 10^3^ cells per well in 100-mm dishes) treated with DMSO or t-BHQ (10 *μ*M) were incubated in basal growth medium for 12 days. The colony-forming abilities were compared between EP-MSCs treated with DMSO or t-BHQ using CV staining, and the numbers of colony-forming cells were counted in triplicate by three observers (*n*=3). *P<0.05 compared with DMSO-treated EP-MSCs. (**f**) EP-MSCs (8 × 10^4^ cells per well in 12-well plates) treated with DMSO or t-BHQ (10 *μ*M) were incubated in osteogenic medium for 10 days. Alizarin red S staining was performed to detect mineral deposition at day 10. For quantitative analysis, absorbance was measured at 595 nm following destaining with 10% cetylpyridinium for 30 min. *P<0.05 compared with DMSO-treated EP MSCs

*tert*-Butylhydroquinone (t-BHQ) is an activator of NRF2 and regulates NRF2 stabilization by preventing binding with KEAP1.^27^ We treated EP MSCs with 10 *μ*M t-BHQ in order to evaluate the self-renewal and osteogenic differentiation potentials. We observed an increase in NRF2 phosphorylation in the nucleus when EP MSCs were exposed to t-BHQ at a dose of 10 *μ*M for 12 h (Figure 3a). It was difficult to observe the difference in the nucleic NRF2 protein levels between control EP MSCs and t-BHQ-treated EP MSCs using immunofluorescence because nucleic NRF2 was generally abundant (Figure 3b). Nevertheless, the mRNA levels of *HO-1* and *NQO-1* were significantly higher in t-BHQ-treated EP MSCs than in control cells (Figure 3c). t-BHQ enhanced the proliferation rate and increased the number of colony-forming cells in t-BHQ-treated EP-MSCs compared with control EP-MSCs, as expected (Figures 3d and e). Unexpectedly, the osteogenic differentiation of EP MSCs was blocked by 10 *μ*M t-BHQ (Figure 3f). In general, pluripotent or stemness genes, such as *SOX2*, *NANOG*, and *OCT3/4*, enable the maintenance of self-renewal and undifferentiated states in stem cells.^28^ Therefore, we inferred that the inhibition of osteogenic differentiation caused by NRF2 activation in EP MSCs was due to NRF2-mediated stemness gene regulation.

### Enhanced NRF2 activity maintains the undifferentiated state of EP MSCs by increasing SIRT1 expression at the mRNA and protein levels

**Figure 4:**
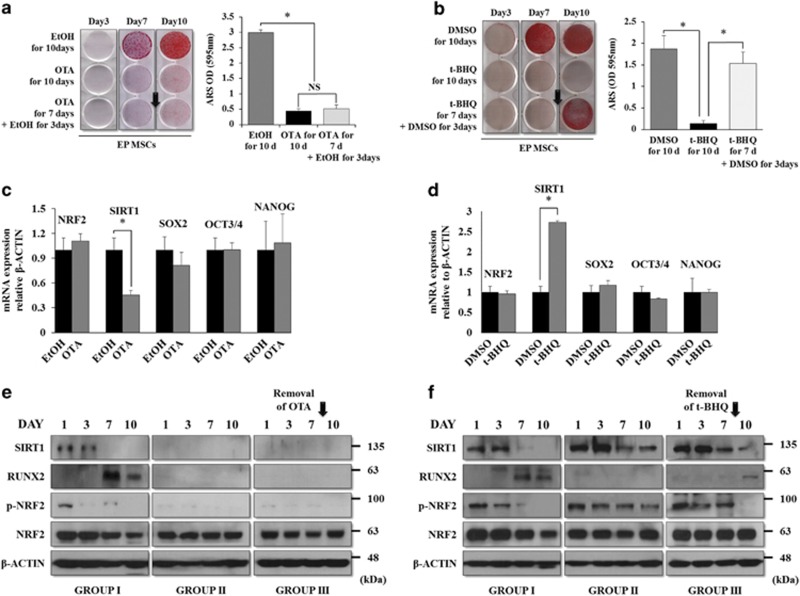
Rescue patterns of suppressed osteogenic potential upon the removal of OTA or t-BHQ, and the association with NRF2 nuclear localization and stemness genes. (**a**) EP MSCs (8 × 10^4^ cells per well in 12-well plates) treated with EtOH or OTA (10 *μ*M) were incubated in osteogenic medium for 10 days. In addition, OTA application was stopped on day 7 of osteogenic differentiation in another group. Alizarin red S staining was performed to detect mineral deposition at days 3, 7, and 10. For quantitative analysis, absorbance was measured at 595 nm following destaining with 10% cetylpyridinium for 30 min. *P<0.05 compared with EtOH- or OTA-treated EP MSCs. (**b**) Similarly, EP MSCs (8 × 10^4^ cells per well in 12-well plates) treated with DMSO or t-BHQ (10 *μ*M) were incubated in osteogenic medium for 10 days. In addition, t-BHQ application was stopped on day 7 of osteogenic differentiation in another group. Alizarin red S staining was performed to detect mineral deposition at days 3, 7, and 10. *P<0.05 compared with DMSO- or t-BHQ-treated EP MSCs. (**c** and **d**) EP MSCs were incubated in basal growth medium (DMEM-LG containing 10% FBS) in the presence of EtOH/DMSO or OTA (10 *μ*M)/t-BHQ (10 *μ*M) for 16 h. The mRNA expression levels of *NRF2*, *SIRT1*, *SOX2*, *OCT3/4*, and *NANOG* were also analyzed by qRT-PCR. *P<0.05 compared with control EtOH/DMSO-treated MSCs. (**e** and **f**) EtOH/DMSO or OTA (10 *μ*M)/t-BHQ (10 *μ*M)-treated EP MSCs differentiated to the osteogenic lineage, and cell pellets in each condition were harvested at each stage to prepare cell lysates. The protein levels of NRF2, phosphorylated NRF2, RUNX2, and SIRT1 were analyzed by western blot analysis. The protein level of *β*-CATENIN was used as a loading control

Three groups were examined. The first group (GROUP I) was EP-MSCs grown for 10 days in osteogenic induction medium with each vehicle for OTA or t-BHQ. The second group (GROUP II) was EP-MSCs grown for 10 days in osteogenic induction medium with OTA or t-BHQ at a dose of 10 *μ*M. The third group (GROUP III) was EP-MSCs grown for 7 days in an osteogenic induction medium with OTA or t-BHQ at a dose of 10 *μ*M. The cells were grown for an additional 3 days in osteogenic induction medium without OTA or t-BHQ. As shown in Figure 4a, OTA-treated EP MSCs did not differentiate to the osteogenic lineage. The removal of OTA on day 7 of differentiation did not rescue the osteogenic potential of EP MSCs. t-BHQ-treated EP MSCs also did not differentiate to the osteogenic lineage. However, the removal of t-BHQ on day 7 significantly rescued the osteogenic potential (Figure 4b). Next, we performed quantitative real-time PCR (qRT-PCR) to confirm the regulation of stemness genes by NRF2. As mentioned previously, *SOX2*, *NANOG*, and *OCT3/4* are very important stemness genes for the maintenance of MSC self-renewal and multipotency,^4, 29, 30, 31^ and *SIRT1* is also thought to regulate MSC stemness.^32^ The regulation of NRF2 activity by OTA or t-BHQ treatment in EP MSCs did not affect the mRNA expression of *SOX2*, *OCT4*, or *NANOG*. Among the stemness genes, only *SIRT1* expression was significantly affected by OTA or t-BHQ treatment (Figures 4c and d). We previously reported that among the general stemness genes, SOX2 may be a key factor for maintaining self-renewal and multipotency. Moreover, SOX2 is regulated via post-translational modification such as acetylation in MSCs, and SIRT1, a lysine deacetylase, directly modulates SOX2 through the inhibition of SOX2 degradation via ubiquitination, although the mRNA level of *SOX2* is not changed.^32^ Thus, we selected *SIRT1* as a candidate gene that may be regulated by NRF2 activity. We examined whether the t-BHQ-mediated interference of osteogenic differentiation was due to the maintenance of the undifferentiated state of EP MSCs by *SIRT1*. The p-NRF2 and SIRT1 protein levels decreased, whereas the protein level of Runt-related transcription factor 2 (RUNX2), a master transcription factor involved in osteogenic differentiation, increased during the osteogenic differentiation process (GROUP I) (Figure 4e, left). For the sustained OTA treatment during the osteogenic differentiation of EP MSCs (GROUP II), p-NRF2, SIRT1, and RUNX2 were not observed during the differentiation processes (Figure 4e, middle). After the removal of OTA on day 7 of the differentiation process, p-NRF2, SIRT1, and RUNX2 expression levels were not rescued (Figure 4e, right). Sustained t-BHQ treatment maintained the protein levels of p-NRF2 and SIRT1, whereas the p-NRF2 and SIRT1 protein levels decreased and the RUNX2 protein level increased after the removal of t-BHQ on day 7 of the differentiation process (Figure 4f). These results suggest that NRF2 localization or activity is an important regulator in the SIRT1-mediated maintenance of MSC stemness. In this experiment, the inhibition of NRF2 activity by OTA induced the loss of the self-renewal and osteogenic differentiation potential, whereas NRF2 activation by t-BHQ enhanced the self-renewal and osteogenic differentiation potentials of EP MSCs. Therefore, the cellular localization of NRF2 in MSCs may be a crucial factor that determines stem cell characteristics.

### p53 is a negative mediator of NRF2-induced SIRT1 stabilization

**Figure 5:**
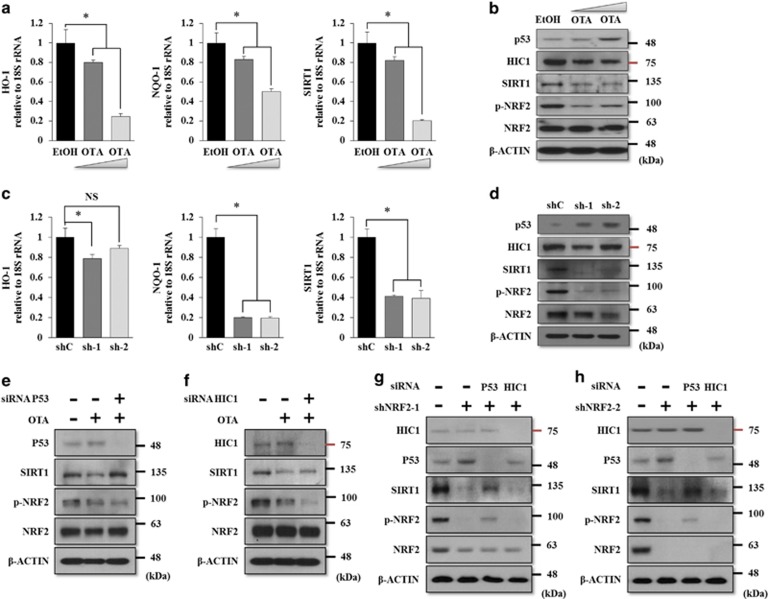
NRF2-mediated SIRT1 regulation occurs in a p53-dependent manner in EP MSCs. (**a**) EP MSCs were incubated in basal growth medium (DMEM-LG containing 10% FBS) in the presence of EtOH, 1 *μ*M OTA, and 10 *μ*M OTA for 16 h. The mRNA expression levels of *HO-1*, *NQO-1*, and *SIRT1* were also analyzed by qRT-PCR. *P<0.05 compared with control EtOH-treated MSCs. (**b**) The protein levels of NRF2, phosphorylated NRF2, SIRT1, HIC1, and p53 were analyzed by western blot analysis. The protein level of *β*-CATENIN was used as a loading control. (**c**) The mRNA expression levels of *HO-1*, *NQO-1*, and *SIRT1* were also analyzed in EP MSCs infected with nontargeting shRNA, shRNF2-1, or shNRF2-2 by qRT-PCR. *P<0.05 compared with nontargeting shRNA-infected MSCs. (**d**) Similarly, the protein levels of NRF2, phosphorylated NRF2, SIRT1, HIC1, and p53 were analyzed in EP MSCs infected with nontargeting shRNA, shRNF2-1, or shNRF2-2 by western blot analysis. (**e**) siRNA targeting p53 was transfected in the OTA-treated EP MSCs. The protein levels of NRF2, phosphorylated NRF2, SIRT1, and p53 were analyzed in EtOH-, OTA (10 *μ*M)-, or siRNA targeting p53 plus OTA (10 *μ*M)-treated EP MSCs. (**f**) siRNA targeting HIC1 was transfected in the OTA-treated EP MSCs. The protein levels of NRF2, phosphorylated NRF2, SIRT1, and HIC1 were analyzed in EtOH-, OTA (10 *μ*M)-, or siRNA targeting HIC1 plus OTA (10 *μ*M)-treated EP MSCs. (**g**) siRNA targeting p53 or HIC1 was transfected with shNRF2-1-infected EP MSCs. The protein levels of NRF2, phosphorylated NRF2, SIRT1, HIC1, and p53 were analyzed in the EP MSCs with nontargeting shRNA, shNRF2-1 alone, shNRF2-1 plus siRNA targeting p53, or shNRF2-1 plus siRNA targeting HIC1. (**h**) siRNA targeting p53 or HIC1 was transfected with shNRF2-2-infected EP MSCs. The protein levels of NRF2, phosphorylated NRF2, SIRT1, HIC1, and p53 were analyzed in the EP MSCs with nontargeting shRNA, shNRF2-2 alone, shNRF2-2 plus siRNA targeting p53, or shNRF2-2 plus siRNA targeting HIC1. The protein level of *β*-CATENIN was used as a loading control. NS, no significance; shC, nontargeting shRNA; sh-1, shNRF2-1; sh-2, shNRF2-2

*SIRT1* transcription is negatively regulated by p53 and hypermethylated cancer 1 (HIC1).^33^ HIC1 directly represses SIRT1 transcription via binding the *SIRT1* promoter.^34^ The *SIRT1* promoter also contains two p53 binding sites, and p53 functions as a transcriptional repressor of SIRT1.^35^ Based on these results, we hypothesized that the decrease in the SIRT1 protein levels by the inhibition of NRF2 activity is due to an increase in HIC1 or p53. NRF2 induces the degradation of tumor-suppressor genes, such as *p53* or *HIC1*, through direct interactions between tumor-suppressor genes and NRF2 downstream target genes.^36, 37^ We confirmed that SIRT1 was transcriptionally regulated by the inhibition of activity or the knockdown of *NRF2*. As shown in Figure 5a, OTA treatment at doses of 1 or 10 *μ*M reduced the mRNA levels of *HO-1*, *NQO-1*, and *SIRT1* dose dependently in EP MSCs. The western blot results also showed that OTA treatment significantly decreased the protein levels of p-NRF2 and SIRT1. In contrast, the p53 protein level, but not HIC1, was highly increased by OTA treatment in EP MSCs (Figure 5b). Using small hairpin RNA (shRNA) targeting NRF2, the mRNA expression levels of *HO-1*, *NQO-1*, and *SIRT1* decreased significantly in shNRF2-infected EP MSCs (Figure 5c). NRF2 knockdown in EP MSCs induced a decrease in the SIRT1 protein level and an increase in the p53 protein level, but did not affect HIC1 (Figure 5d). These results imply that p53 induced by NRF2 inhibition may suppress the level of SIRT1 expression in EP MSCs. To verify this hypothesis, we purchased siRNA targeting the human *p53* or *HIC1* genes. In the western blot analysis, OTA decreased the protein levels of p-NRF2 and SIRT1, and increased the p53 protein level. In the same conditions, *p53* knockdown using siRNA rescued the SIRT1 protein level, even though the p-NRF2 protein level was still decreased in these OTA-treated EP MSCs (Figure 5e). Knockdown of *HIC1* did not affect the SIRT1 protein level in the same conditions (Figure 5f). Knockdown of *NRF2* using the shRNA system also successfully decreased the protein levels of NRF2, p-NRF2, and SIRT1, and increased the p53 protein level, but did not affect HIC1. Knockdown of p53 in NRF2-targeting shRNA-infected EP MSCs also rescued the SIRT1 protein level (Figure 5h). These results indicate that p53 is involved in the NRF2-mediated SIRT1 regulation, whereas SIRT1 regulation via HIC1 was not observed. Thus, SIRT1 regulation is predominantly affected by p53, rather than HIC1, in human MSCs.

### The self-renewal and osteogenic differentiation potentials suppressed by the inhibition of NRF2 activity can be rescued by p53 RNA interference

**Figure 6:**
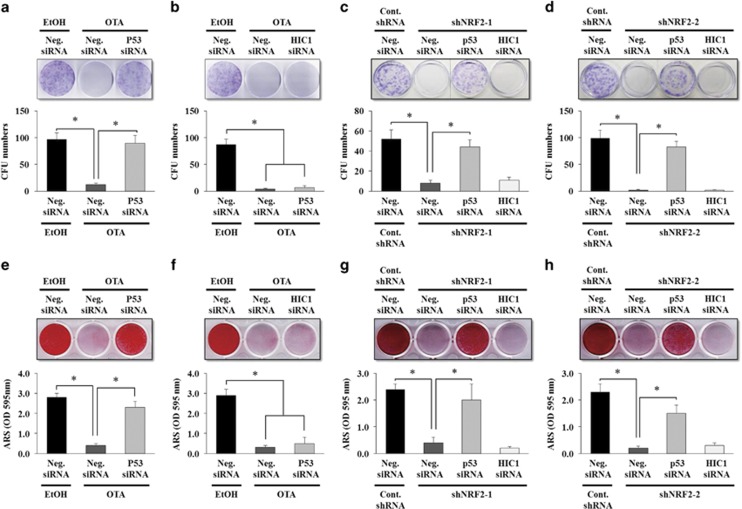
RNA interference of p53 rescues the self-renewal and osteogenic potentials that are suppressed in OTA-treated and shNRF2-infected EP MSCs. The siRNAs targeting p53 (**a**) or HIC1 (**b**) were transfected in EP MSCs, and the cells were plated at 1 × 10^3^ cells per well in 100-mm dishes. After 24 h, OTA (10 *μ*M) was treated with the siRNA-transfected cells, and the cells were incubated in basal growth medium for 12 days. After 12 days, CV staining was performed, and the numbers of colony-forming cells were counted in triplicate by three observers (*n*=4). *P<0.05 compared with EtOH-treated EP MSCs transfected with negative siRNA or OTA-treated EP MSCs transfected with negative siRNA. Similarly, the siRNAs targeting p53 or HIC1 were transfected in shNRF2-1 (**c**)- or shNRF2-2 (**d**)-infected EP MSCs, and the cells were plated at 1 × 10^3^ cells per well in 100 mm dishes and incubated in basal growth medium for 12 days. After 12 days, CV staining was performed, and the numbers of colony-forming cells were counted in triplicate by three observers (*n*=3). *P<0.05 compared with nontargeting shRNA-infected EP MSCs transfected with negative siRNA or shNRF2-infected EP-MSCs transfected with negative siRNA. The siRNAs targeting p53 (**e**) or HIC1 (**f**) were transfected in EP MSCs, and the cells were plated at 8 × 10^4^ cells per well in 12-well plates. After 24 h, OTA (10 *μ*M) was treated with the siRNA-transfected cells, and the cells were incubated in osteogenic medium for 10 days. After 10 days, alizarin red S staining was performed to detect mineral deposition. For quantitative analysis, absorbance was measured at 595 nm following destaining with 10% cetylpyridinium for 30 min. *P<0.05 compared with EtOH-treated EP MSCs transfected with negative siRNA or OTA-treated EP MSCs transfected with negative siRNA (*n*=4). Similarly, the siRNAs targeting p53 or HIC1 were transfected in shNRF2-1 (**g**)- or shNRF2-2 (**h**)-infected EP MSCs, and the cells were incubated in osteogenic medium for 10 days. After 10 days, alizarin red S staining was performed to detect mineral deposition. For quantitative analysis, absorbance was measured at 595 nm following destaining with 10% cetylpyridinium for 30 min (*n*=3). *P<0.05 compared with nontargeting shRNA-infected EP MSCs transfected with negative siRNA or shNRF2-infected EP MSCs transfected with negative siRNA. Cont.shRNA, nontargeting shRNA; Neg.siRNA, nontargeting siRNA

We further examined whether targeting p53 would rescue the suppressed self-renewal and differentiation potentials in OTA-treated or shNRF2-infected EP MSCs. Figure 6a shows that siRNA targeting p53 rescued the colony-forming ability that was decreased in OTA-treated EP MSCs, but this effect was not observed for siRNA targeting HIC1 (Figures 6a and b). As expected, in the shNRF2-infected EP MSCs, siRNA targeting p53 significantly enhanced the colony-forming ability that was suppressed by shRNA targeting NRF2, whereas targeting HIC1 did not affect the self-renewal capacity (Figures 6c and d). The osteogenic potential suppressed by OTA treatment was also rescued by siRNA targeting p53. Alizarin red S staining showed that suppressed calcification by OTA treatment was rescued in p53-targeting siRNA-transfected EP MSCs (but not in HIC1-targeting siRNA-transfected EP MSCs) (Figures 6e and f). Similarly, in shNRF2-infected EP MSCs, targeting p53 using siRNA yielded the same results to those of the CFU-F assay and alizarin red S staining (Figures 6g and h). Accordingly, p53 may be an important target in NRF2-mediated SIRT1 regulation for the maintenance of the self-renewal and osteogenic differentiation capacities during prolonged MSC culture.

### The self-renewal and osteogenic potentials of LP MSCs can be reactivated by t-BHQ treatment

**Figure 7:**
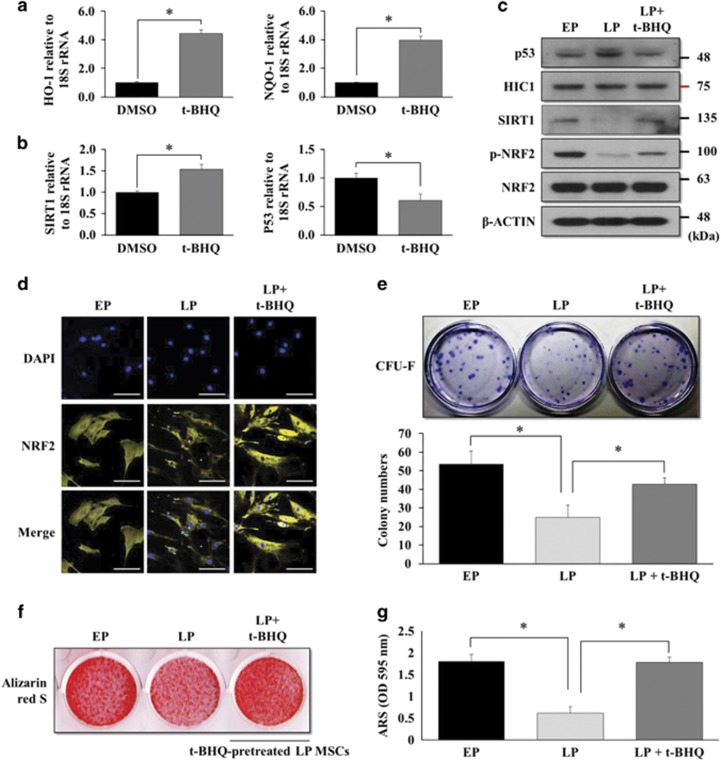
NRF2 activation by t-BHQ enhances the self-renewal capacity and osteogenic potential of LP MSCs. (**a**) LP MSCs were grown in basal growth medium (DMEM-LG containing 10% FBS) in the presence of t-BHQ (10 *μ*M) for 7 days. The mRNA expression levels of *HO-1*, *NQO-1*, (**b**) *SIRT1*, and *p53* were also analyzed by qRT-PCR. *P<0.05 compared with control EtOH-treated MSCs. (**c**) Similarly, LP MSCs were grown in basal growth medium in the presence of t-BHQ for 7 days before the western blot analysis. The protein levels of NRF2, phosphorylated NRF2, SIRT1, HIC1, and p53 were analyzed, and the protein level of *β*-CATENIN was used as a loading control. (**d**) Immunofluorescence was performed to observe the nuclear and cytosolic localization of NRF2 in the EP or LP MSCs or t-BHQ- (10 *μ*M)-pretreated LP MSCs. The nuclei were stained with DAPI, and NRF2 was stained with Alexa Fluor 568 (Yellow)-conjugated secondary antibody. The images were obtained using confocal microscopy. Scale bar=100 *μ*m. (**e**) EP MSCs, LP MSCs, or t-BHQ-pretreated LP MSCs were incubated in basal growth medium for 12 days. The colony-forming abilities were compared using CV staining, and the numbers of colony-forming cells were counted in triplicate by three observers (*n*=4). *P<0.05 compared with EP or LP MSCs. (**f**) Before the osteogenic differentiation of LP MSCs, LP MSCs were pretreated with t-BHQ. At the starting point of differentiation, LP MSCs were not treated with t-BHQ. EP MSCs, LP MSCs, or t-BHQ-pretreated LP MSCs were plated at 8 × 10^4^ cells per well in 12-well plates and maintained in osteogenic medium for 10 days. After 10 days, alizarin red S staining was performed to detect mineral deposition. (**g**) For quantitative analysis, absorbance was measured at 595 nm following destaining with 10% cetylpyridinium for 30 min. *P<0.05 compared with EP or LP MSCs

Finally, we tested whether the decreased self-renewal and osteogenic potentials of LP MSCs can be reactivated by inducing the nuclear import of cytosolic NRF2. Application of t-BHQ to LP MSCs increased the mRNA expression of *HO-1*, *NQO-1*, and *SIRT1*, and decreased mRNA expression of *p53* (Figures 7a and b). We also found that the p53 protein level was higher in LP-MSCs than EP MSCs, but the HIC1 protein level was not affected by prolonged cell passages. t-BHQ treatment in LP MSCs increased the protein level of p-NRF2, resulting in decreased p53 and increased SIRT1 (Figure 7c). We also confirmed the nuclear localization of NRF2 in t-BHQ-treated LP MSCs (Figure 7d). The self-renewal and multipotency of MSCs decreased during prolonged cell passages. Surprisingly, reactivating the NRF2–SIRT1 axis by applying t-BHQ to LP MSCs enhanced the colony-forming ability of the cells (Figure 7e). Furthermore, LP MSCs pretreated with t-BHQ for ∼4–7 days before osteogenic differentiation were successfully differentiated to the osteogenic lineage (Figures 7f and g). These results suggest that the NRF2–SIRT1 axis is an important target in the maintenance of the self-renewal and differentiation potencies of MSCs as well as in rejuvenating long-term MSC cultures *in vitro* before animal studies and clinical applications.

## Discussion

Our study showed that the inhibition or induction of NRF2 nuclear localization affected cell proliferation and the colony-forming ability of EP MSCs. Interestingly, the *in vitro* osteogenic potential of MSCs was decreased by treatment with both t-BHQ (a NRF2 activator) and OTA (a NRF2 inhibitor). These results indicate that NRF2 activity is essential for maintaining the self-renewal and osteogenic potentials of MSCs during *ex vivo* cultivation. Because the sustained nuclear localization of NRF2 also blocked MSC differentiation to the osteogenic lineage, it is possible that sustained nuclear NRF2 maintains an undifferentiated state of MSCs; the undifferentiated state can be maintained in hypoxic environments, mimicked by NRF2/hypoxia-inducible factor-1*α* (HIF-1*α*) signaling.^12, 38^ Furthermore, oxidative stress induces cellular senescence of human MSCs.^39^ Accordingly, we suggest that sustained NRF2 activation protects MSCs against oxidative stress during *ex vivo* cultivation,^39^ resulting in the maintenance of MSC stemness. NRF2 negatively regulates osteogenic differentiation^40^ as well as osteoclastic differentiation.^41^ In addition, there are conflicting results regarding the role of NRF2 in osteogenic differentiation. Human periodontal ligament cells can be efficiently differentiated to the osteogenic lineage by increasing NRF2 levels in nuclear extracts.^42^ NRF2-knockout mice show a significant deficit in postnatal bone acquisition.^43^ Taken together, NRF2 is a key factor in MSC maintenance and osteogenesis; when NRF2 is lacking, MSCs cannot self-renew and differentiate to the osteogenic lineage.

NRF2 also has important roles in the chondrogenic and adipogenic differentiation of MSCs. NRF2 is a negative regulator of cellular differentiation in a chondrocytic cell line, ATDC5. NRF2 overexpression decreases mRNA expression of type II collagen in ATDC5 cells.^44^ However, sulforaphane (SFN), another NRF2 activator, can suppress gene expression related to osteoarthritis and block cartilage destruction.^45^ The protein level of nuclear Nrf2 also continuously decreases during adipogenic differentiation in ST2 cells, a bone marrow-derived MSC cell line.^46^ This expression pattern is required for normal adipocyte differentiation of 3T3L1 cells and is associated with increased oxidative stress levels that can facilitate differentiation processes.^47^ SFN can also suppress the adipogenic differentiation of 3T3L1 cells.^48^ In contrast, OTA inhibits the adipogenic differentiation of adipose-derived MSCs.^49^ This is consistent with our results regarding the osteogenic differentiation of MSCs. Taken together, NRF2 may be indispensible for the maintenance of the MSC self-renewal capacity as well as the multi-differentiation potential to the osteogenic, chondrogenic, and adipogenic lineages.

**Figure 8:**
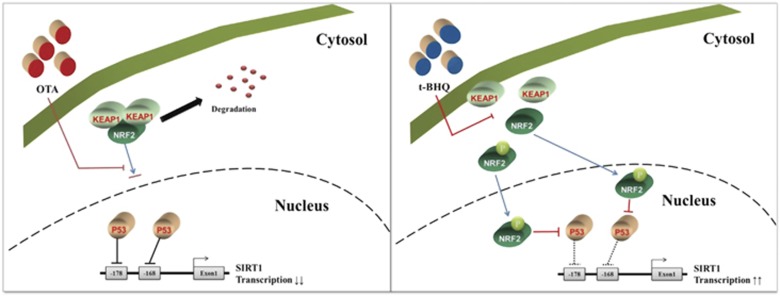
Proposed models of the effects of OTA or t-BHQ on the regulation and maintenance of MSC stemness via SIRT1. Blocking the nuclear import of NRF2 activates p53, which suppresses SIRT1 promoter activity, resulting in a loss of MSC stemness. Conversely, the protein level of p53 can be decreased by the phosphorylation and nuclear import of NRF2, resulting in the activation of SIRT1 transcription as well as the enhancement of MSC stemness

SIRT1 has been a recent focus of research as a regulator of MSC stemness.^50^ It is required for the long-term cultivation of MSCs,^51^ and its overexpression ameliorates cellular senescence by reversing aged MSCs, resulting in an increase in cell proliferation.^52^ SIRT1 regulates the osteogenic and chondrogenic differentiation of MSCs by deacetylating *β*-catenin.^53^ For the maintenance of stemness, SIRT1 positively regulates the self-renewal and multipotency of human MSCs by deacetylating the lysine residue of SOX2.^32^ SIRT1 is known to induce deacetylation of NRF2 that subsequently decreases NRF2-dependent gene transcription.^54^ However, it is thought that NRF2 and SIRT1 have similar functions in adult stem cells because the loss of NRF2 in the intestinal stem cells of *Drosophila* induces ROS levels and age-related degeneration.^55^ Similarly, the loss of SIRT1 is also related to an increase in ROS levels in hematopoietic stem and progenitor cells.^56^ These reports indicate that the interaction between NRF2 and SIRT1 in adult stem cells may be involved in defense against oxidative stress during prolonged cell passages, preventing cellular aging and the loss of multipotency. p53 can directly bind to two *SIRT1* promoter regions and suppress the transcriptional activity of SIRT1,^35^ whereas NRF2 can induce the degradation of p53 via the regulation of *Mdm2* expression.^57^ Based on these results, our study showed that the p53 protein level increased and the SIRT1 protein level decreased in OTA-treated or shNRF2-infected MSCs, and RNA interference of p53 in OTA-treated or shNRF2-infected MSCs rescued the SIRT1 protein level as well as the osteogenic differentiation potential. Furthermore, t-BHQ rejuvenated LP MSCs by enhancing the SIRT1 expression level via NRF2-mediated p53 suppression (Figure 8). Given the importance of these findings in MSCs, additional studies will further increase our understanding of the role of NRF2 in MSC stemness and will facilitate the optimization of the application of NRF2 activators to MSCs.

Recent studies have reported the mechanism of NRF2 action and its new function, excluding its well-known role in antioxidant response. Here, we reviewed the mechanism of NRF2 action that has been reported in several types of stem cells (Supplementary Table S1). In embryonic stem cells, NRF2 acts as a regulator of the proteasome that regulates self-renewal and pluripotency.^25^ In addition, NRF2 can modulate self-renewal and quiescence by regulating CXCR4 in hematopoietic stem cells.^58^ Our study was the first to clarify the molecular mechanisms involved in the regulation of MSCs by NRF2 to maintain the self-renewal and osteogenic potentials via SIRT1. Our findings provide mechanistic insight into how NRF2 contributes to the maintenance of MSC stemness. Thus, the conservation of NRF2 nuclear localization is an important target for the prevention of cellular senescence and the loss of multipotency during prolonged cell passage of *ex vivo* cultured MSCs.

## Materials and Methods

### Cell culture, differentiation, and drug treatment

Bone marrow aspirates were obtained from the posterior iliac crests of seven adult donors, with the approval of the institutional review board (IRB) of the Yonsei University College of Medicine. MSCs isolated from bone marrow were selected based on their ability to adhere to plastic cell culture dishes, and >98% of the cultured cells were positive for CD90 and CD105, but negative for CD34 and CD45,^59^ as previously described. MSCs were maintained in low-glucose Dulbecco's modified Eagle's medium (DMEM-LG; Invitrogen, Carlsbad, CA, USA) supplemented with 10% fetal bovine serum (FBS; Gibco, Grand Island, NY, USA) and 1% antibiotic–antimycotic solution (Invitrogen) at 37 °C in a 5% CO_2_ atmosphere. EP MSCs (passages 1–3) were replated at a density of 5000 cells/cm^2^, and the cells were subcultured when they were 80% confluent up to passages 7–10 (LP MSCs). To induce osteogenic differentiation, MSCs were seeded onto 12-well culture pates at a density of 8 × 10^4^ cells per well. The medium used for the osteogenic differentiation of MSCs has been described previously.^5^ Alizarin red S staining was used to evaluate osteogenic differentiation. Briefly, after cells were fixed in ice-cold 70% ethanol, freshly prepared 3% alizarin red S solution (wt/vol) (Sigma, St. Louis, MO, USA) was added, and cells were incubated in the dark for 30 min. For quantitative analysis of alizarin red S, absorbance was detected at 595 nm following destaining with 10% cetylpyridinium chloride monohydrate (Sigma) for 20 min. OTA (Sigma) was dissolved in ethanol (EtOH) and used at a concentration of 1–10 *μ*M in EP MSCs. t-BHQ (Sigma) was dissolved in dimethyl sulfoxide (DMSO) and used at a concentration of 1–10 *μ*M in EP or LP MSCs.

### Quantitative real-time PCR (qRT-PCR)

Real-time PCR analysis was performed as described previously.^32^ Briefly, total RNA was isolated using an RNeasy Kit (Qiagen, Valencia, CA, USA), according to the manufacturer's instructions. Total RNA (1 *μ*g) was reverse-transcribed using the Omniscript Kit (Qiagen). Primer sets were validated and purchased from Bioneer (Daejeon, South Korea; http://www.bioneer.co.kr/). The primers used were as follows: *NRF2* (P164742), *HO-1* (P133045), *NQO-1* (P113225), *SIRT1* (P293039), *SOX2* (P200205), and *NANOG* (P255522). There are no validated primers for *OCT3/4(a)* or *β-ACTIN*. To obtain PCR products specific to *OCT3/4(a)*, which acts as a transcription factor, the primer must include exon 1 in its recognition site;^60^ thus, the following primers were designed: 5′-GCAAGCCCTCATTTCACCA-3′ (sense, NM_002701) and 5′-GCCCATCACCTCCACCAC-3′ (antisense). The following primers were designed for *β-ACTIN*: 5′-GTCCTCTCCCAAGTCCACACA-3′ (sense, NM_001101.3) and 5′-GGGCACGAAGGCTCATCATTC-3′ (antisense). Mean cycle threshold values from triplicate (*n*=3) measurements were used to calculate gene expression, with normalization to *β-ACTIN* as an internal control.

### Western blot analysis

MSCs were lysed in passive lysis buffer (Promega, Madison, WI, USA). Protein concentrations were determined using the Bio-Rad Protein Assay (Bio-Rad Laboratories, Inc., Hercules, CA, USA) and 30 mg of protein was analyzed by 10% sodium dodecyl sulfate-polyacrylamide gel electrophoresis (SDS-PAGE) (Sigma). Transferred membranes were blocked with 5% skim milk (BD, Sparks, MD, USA), and incubated for 10 h with antibodies against NRF2 (Santa Cruz Biotechnology, Santa Cruz, CA, USA), phosphorylated-NRF2 (Abcam, Cambridge, UK), HDAC1 (Santa Cruz Biotechnology), HSP90 (Santa Cruz Biotechnology), LAMIN-B (Santa Cruz Biotechnology), LDH (Santa Cruz Biotechnology), RUNX2 (EMD Millipore, San Diego, CA, USA), SIRT1 (Santa Cruz Biotechnology), p53 (Santa Cruz Biotechnology), and HIC1 (Santa Cruz Biotechnology). Membranes were further probed with an antibody against *β*-*ACTIN* (Santa Cruz Biotechnology, Dallas, TX, USA) that served as a loading control.

### Nuclear and cytosolic fractionation

EP, LP, undifferentiated, or differentiated MSCs were collected by trypsinization and washed with phosphate-buffered saline (PBS) two times before nuclear and cytosolic fractionation. Nuclear and cytoplasmic fractionation were conducted using the NE-PER Nuclear and Cytoplasmic Extraction Reagents Kit (Thermo Fisher Scientific, Rockford, IL, USA) according to the manufacturer's instructions. Each separated protein was analyzed by western blot analysis.

### Immunocytochemistry

EP, LP, undifferentiated, differentiated, OTA-, or t-BHQ-treated MSCs were seeded at 5000 cells/cm^2^ on 4-well glass chamber slides (Nalge Nunc International, Rochester, NY, USA), and the cells were incubated in a 5% CO_2_ incubator at 37 °C. After an overnight incubation, the cells were washed with PBS followed by fixation with 4% paraformaldehyde (Sigma) for 30 min. Permeabilization was accomplished with 1% Triton X-100 in PBS for 10 min followed by blocking for 1 h with 3% bovine serum albumin (BSA) in PBS. The cells were incubated with a 1 : 200 dilution of primary antibodies against NRF2 (Santa Cruz Biotechnology) overnight at 4 °C. After washing three times with PBS, the cells were incubated with fluorescein isothiocyanate (FITC) and phycoerythrin-conjugated secondary antibodies (Santa Cruz Biotechnology) or Alexa Fluor 568 (Yellow, Abcam) in a 1 : 5000 dilution in 3% BSA-containing PBS for 1 h at room temperature in the dark. The nuclei were stained with 4,6-diamidino-2-phenyindole (DAPI, Sigma) and then examined using a Zeiss LSM700 scanning laser confocal microscope (Zen 2011; Carl Zeiss MicroImaging GHBH, Jena, Germany).

### Proliferation assay

Cell proliferation was examined using an EZ-Cytox Kit (Daeil Lab Service, Seoul, Korea). OTA- or t-BHQ-treated EP-MSCs were seeded in 12-well culture plates at a density of 1 × 10^4^ cells per well. The cells were maintained in DMEM-LG for 7 days, and the cell culture media were replaced once a day during cell viability assay periods. Briefly, after washing cells in PBS, 10 *μ*l of EZ-Cytox (tetrazolium salts) solution was added to each well and incubated at 37 °C for 4 h. After incubation, the conditioned medium was transferred to 96-well plates. The absorbance was measured at 450 nm. All samples were tested in triplicate (*n*=6).

### CFU-F assay

EP, LP, OTA-, or t-BHQ-treated MSCs were seeded at 1 × 10^3^ cells in 100 mm culture dishes, and maintained in DMEM-LG supplemented with 20% FBS for 10–12 days. OTA-or t-BHQ-treated media were replaced every 2 days during colony formation. Subsequently, the cells were fixed in a 1 : 1 acetone/methanol fixative, stained with a 20% crystal violet (CV) solution (Merck, Darmstadt, Germany) for 30 min in the dark, and washed in distilled water. The colony-forming ability of the stained cells was then evaluated and counted.

### Establishment of NRF2-knockdown MSCs

To obtain lentiviral particles with shNRF2, HEK293T cells were seeded in 100 mm culture dishes at a density of 3 × 10^6^ cells per dish. On the next day, the cells were transfected with lentiviral particles with either a nontargeting shRNA expression plasmid (MISSION Plko.1-puro Empty Vector Control Plasmid DNA, Sigma) or two shNRF2 expression plasmids (NFE2L2 MISSION shRNA Stock, TRC numbers: TRCN0000007555 (shNRF-1), TRCN0000007558 (shNRF-2), Sigma) with the Delta 8.9 plasmid (*gag*, *pol*, and *rev* genes) and VSV-G (envelope plasmid, Sigma) using Lipofectamine 2000 (Invitrogen). After 6 h of transfection, the medium was replaced. The shRNA-transfected HEK293T cells were maintained for 2 days, and then the supernatants were collected and stored at −70 °C. To knockdown *NRF2* in EP-MSCs, the cells were seeded in 6-well plates at a density of 5 × 10^4^ cells per well. After 48 h of infection, the medium was replaced with freshly prepared medium with 10 *μ*g/ml puromycin dihydrochloride (Sigma), and the cells were maintained for 7 days. The knockdown efficiency of the selected cells was analyzed by western blot analysis. Below is a list of shRNAs targeting NRF2 used in this study.

1. shNRF2-1 (region: 3′ UTR)

5′-CCGGGCTCCTACTGTGATGTGAAATCTCGAGATTTCACATCACAGTAGGAGCTTTTT-3′

2. shNRF2-2 (region: CDS)

5′-CCGGCCGGCATTTCACTAAACACAACTCGAGTTGTGTTTAGTGAAATGCCGGTTTTT-3′

### siRNA transfection

Scramble, HIC1, and p53 siRNAs were purchased from Bioneer (http://sirna.bioneer.co.kr/). The scramble-sense siRNA targeted the sequence 5′-CCUACGCCACCAAUUUCGU-3′, and the scramble-antisense siRNA targeted the sequence 5′-ACGAAAUUGGUGGCGUAGG-3′. HIC1-sense siRNA targeted the sequence 5′-AGACGAUGCUGGACACGAU (dTdT)-3′, and HIC1-antisense siRNA targeted the sequence 5′-AUCGUGUCCAGCAUCGUCU (dTdT)-3′. p53-sense siRNA targeted the sequence 5′-CACUACAACUACAUGUGUA (dTdT)-3′, and p53-antisense siRNA targeted the sequence 5′-UACACAUGUAGUUGUAGUG (dTdT)-3′. NRF2-sense siRNA targeted the sequence 5′-GAGACUACCAUGGUUCCAA(dTdT)-3′, and NRF2-antisense siRNA targeted the sequence 5′-UUGGAACCAUGGUAGUCUC(dTdT)-3′.

Briefly, EP MSCs treated with OTA (10 *μ*M) or infected with shRNA-1 or shRNA2 were plated to obtain 70–80% confluence in 6-well plates and transfected with 100 nM of HIC1, p53, or scramble (negative control) siRNA using Lipofectamine 2000 (Invitrogen). After 6 h of transfection, fresh medium was exchanged.

### Statistical analysis

Statistical analysis was performed using one-way analysis of variance for multiple comparisons or Student's *t*-tests for differences between two groups, and the data are expressed as means+S.D. Values of *P*<0.05 were considered statistically significant.

## Abbreviations

BM-MSC: bone marrow-mesenchymal stem cell
ROS: reactive oxygen species
NRF2: nuclear factor erythroid-derived 2-like 2
CFU-F: colony-forming unit fibroblast
DAPI: 4,6-diamidino-2-phenyindole
FITC: fluorescein isothiocyanate
t-BHQ: *tert*-butylhydroquinone
HO-1: heme oxygenase-1
NQO-1: NAD(P)H dehydrogenase (quinone 1)
OTA: ochratoxin A
RUNX2: Runt-related transcription factor 2
SIRT1: sirtuin 1
siRNA: small interfering RNA
HIC1: hypermethylated in cancer 1
