# Supplementary figures and images for: Cellular localization of NRF2 determines the self-renewal and osteogenic differentiation potential of human MSCs via the P53–SIRT1 axis
Source: Cell Death Dis. 2016 Feb 11;7(2):e2093–. doi: 10.1038/cddis.2016.3 (PMC4849161; doi:10.1038/cddis.2016.3)

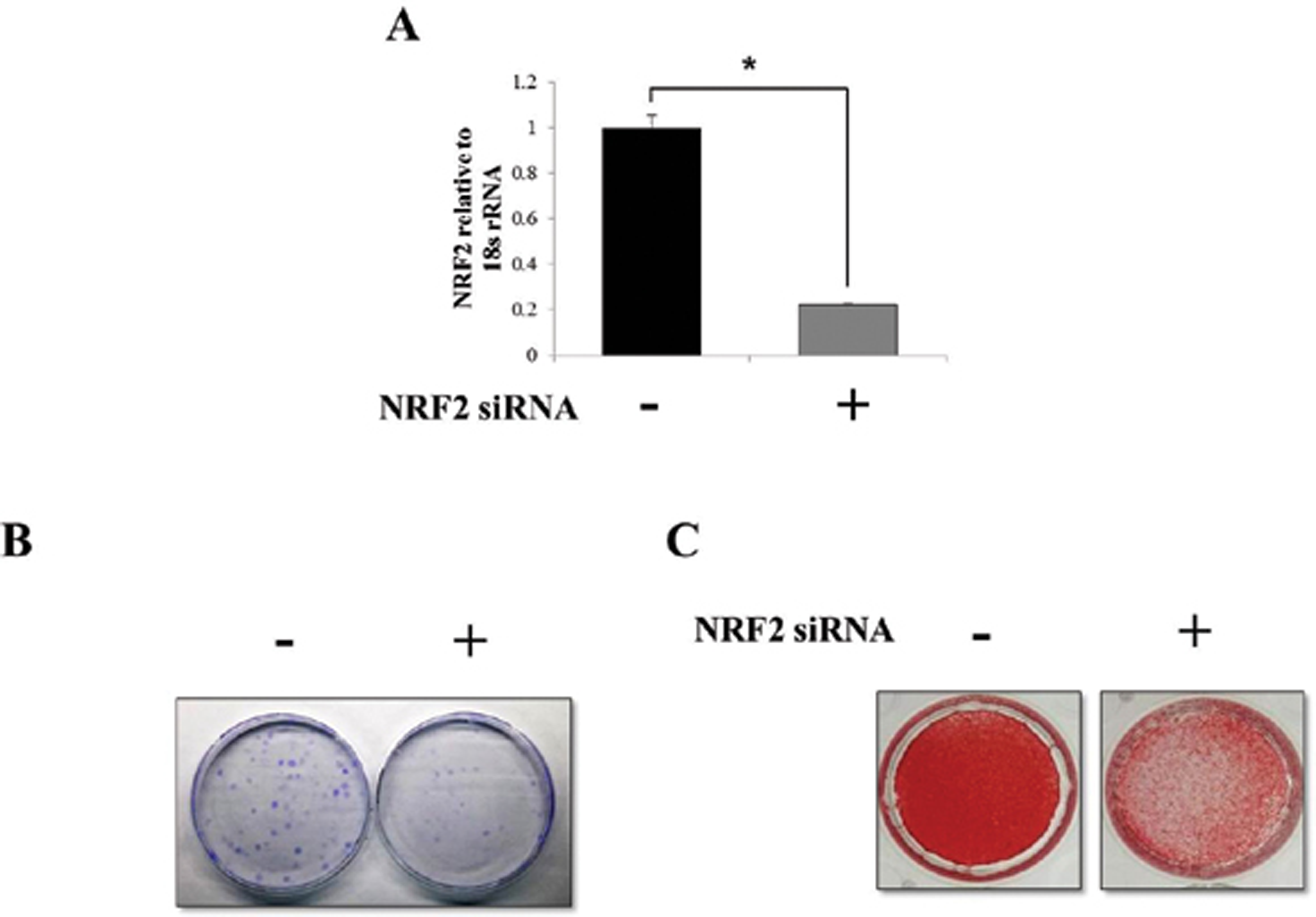

Supplement: Supplementary Figure 1 [file cddis20163x1.tif]
