# Supplementary material for: Cellular localization of NRF2 determines the self-renewal and osteogenic differentiation potential of human MSCs via the P53–SIRT1 axis
Source: Cell Death Dis. 2016 Feb 11;7(2):e2093–. doi: 10.1038/cddis.2016.3 (PMC4849161; doi:10.1038/cddis.2016.3)
Supplement: Supplementary Figure Legends [file cddis20163x2.docx]

**Supplementary Figure S1. Kcockdown of NRF2 via siRNA decreases the self-renewal capacity, osteogenic differentiation and mRNA level of *SIRT1*.**

(A) The mRNA expression levels of *NRF2* in NRF2-knockdown EP-MSCs via siRNA were analyzed by qRT-PCR. (B) NRF2-knockdown EP-MSCs (1 × 10^3^ cells per well in 100-mm dishes) were incubated in basal growth medium for 12 days. The colony-forming abilities were assessed via CV staining. (C) NRF2-Knockdown EP-MSCs (8 × 10^4^ cells per well in 12-well plates) were incubated in osteogenic medium for 10 days. Alizarin red S staining was performed to detect mineral deposition at day 10. *, p < 0.05 compared with control MSCs.
