# Supplementary material for: Cellular localization of NRF2 determines the self-renewal and osteogenic differentiation potential of human MSCs via the P53–SIRT1 axis
Source: Cell Death Dis. 2016 Feb 11;7(2):e2093–. doi: 10.1038/cddis.2016.3 (PMC4849161; doi:10.1038/cddis.2016.3)
Supplement: Supplementary Table 1 [file cddis20163x3.docx]

**Supplementary Table S1. The effects and mechanism of NRF2 in several types of stem cells.**

| **Stem cell type** | **Action** | **Molecular mechanism** | **Effects** | **Reference** |
| --- | --- | --- | --- | --- |
| Embryonic stem cells (ESCs) | NRF2-proteasome pathway | Regulation of OCT4 and NANOG | Self-renewal, differentiation, and cellular reprogramming | *Jang et al. 2014. Stem cells. doi: 10.1002/stem.1764*[^1^](#_ENREF_1) |
| Hematopoietic stem cells (HSCs) | NRF2 over-expression | Regulation of CXCR4 | Self-renewal and quiescence | *Tsai et al. 2013. Nature cell biology. doi: 10.1038/ncb2699*[^2^](#_ENREF_2) |
|  | NRF2 deficiency | Anti-oxidant response | Cell survival and apoptosis | *Merchant et al. Blood. 2011, doi: 10.1182/blood-2011-05-355362*[^3^](#_ENREF_3) |
| Mesenchymal stemc ell (MSCs) | NRF2 over-expression | None | Oxidative stress-induced apoptosis and cytotoxicity | *Mohammadzadeh et al. 2012. Cell stress chaperones. doi: 10.1007/s12192-012-0331-9*[^4^](#_ENREF_4) |
| Glioma stme cells (GSCs) | NRF2 knocodown | Regulation of SOX2 and BMI-1 | Self-renewal | *Zhu et al. 2013. BMC Cancer. doi: 10.1186/1471-2407-13-380*[^5^](#_ENREF_5) |
| Neural stem cells (NSCs) | NRF2 over-expression  and activity | None | Proliferation and neuronal differentiation | *Karkkainen et al. 2014. Ctem cells. dio:10.1002/stem.1666*[^6^](#_ENREF_6) |
| Intestinal stem cells (ISCs) | NRF2-KEAP1 pathway | Redox regulation  by KEAP1 and NRF2 | Cell proliferation | *Hochmuth et al. 2011. Cell stem cell. doi:10.1016/j.stem.2010.12.006*[^7^](#_ENREF_7) |
| Cancer stem cells (CSCs) | NRF2 activity | p62-NRF2 axis | Cell survival and stress resistance | *Oncotarget. 2015 Apr 10; 6(10): 8167–8184*[^8^](#_ENREF_8) |

**REFERENCES**

1. Jang J, Wang Y, Kim HS, Lalli MA, Kosik KS. Nrf2, a regulator of the proteasome, controls self-renewal and pluripotency in human embryonic stem cells. *Stem Cells* 2014; **32**: 2616-2625.

2. Tsai JJ, Dudakov JA, Takahashi K, Shieh JH, Velardi E, Holland AM *et al.* Nrf2 regulates haematopoietic stem cell function. *Nat Cell Biol* 2013; **15**: 309-316.

3. Merchant AA, Singh A, Matsui W, Biswal S. The redox-sensitive transcription factor Nrf2 regulates murine hematopoietic stem cell survival independently of ROS levels. *Blood* 2011; **118**: 6572-6579.

4. Mohammadzadeh M, Halabian R, Gharehbaghian A, Amirizadeh N, Jahanian-Najafabadi A, Roushandeh AM *et al.* Nrf-2 overexpression in mesenchymal stem cells reduces oxidative stress-induced apoptosis and cytotoxicity. *Cell Stress Chaperones* 2012; **17**: 553-565.

5. Zhu J, Wang H, Sun Q, Ji X, Zhu L, Cong Z *et al.* Nrf2 is required to maintain the self-renewal of glioma stem cells. *BMC Cancer* 2013; **13**: 380.

6. Karkkainen V, Pomeshchik Y, Savchenko E, Dhungana H, Kurronen A, Lehtonen S *et al.* Nrf2 regulates neurogenesis and protects neural progenitor cells against Abeta toxicity. *Stem Cells* 2014; **32**: 1904-1916.

7. Hochmuth CE, Biteau B, Bohmann D, Jasper H. Redox regulation by Keap1 and Nrf2 controls intestinal stem cell proliferation in Drosophila. *Cell Stem Cell* 2011; **8**: 188-199.

8. Ryoo IG, Choi BH, Kwak MK. Activation of NRF2 by p62 and proteasome reduction in sphere-forming breast carcinoma cells. *Oncotarget* 2015; **6**: 8167-8184.
